# Supplementary material for: Variation and prognostic potential of the gut antibiotic resistome in the FINRISK 2002 cohort
Source: Nat Commun. 2025 Jul 1;16:5963. doi: 10.1038/s41467-025-61137-x (PMC12214822; doi:10.1038/s41467-025-61137-x)
Supplement: Supplementary file 1 — Supplementary Information [file 41467_2025_61137_MOESM1_ESM.pdf]

# Variation and prognostic potential of the gut antibiotic resistome in the FINRISK 2002 cohort

**Supplementary Table 1: Number of purchases for different antibiotic classes.** Summary of the antibiotic reimbursed purchase events (participants N = 7,095). Data on drug purchases covers seven years before sampling using drug registry data. Antibiotic use is reported using ATC codes for antibiotics. *Purchases* describe the number of reimbursed purchases in the whole Supplementary Data et. *Participants* is the number of participants with reimbursed purchases for the drug. *Mean* and *median* (with range) denote average reimbursed purchases per person.

| | **Purchases (number of events)** | **Participants (N)** | **Mean** | **Standard deviation** | **Median (min-max)** |
|---|---|---|---|---|---|
| Prior antibiotics | 23516 | 5488 | 3.31 | 4.47 | 2 (0-85) |
| Prior non-penicillin betalactams | 5529 | 2667 | 0.78 | 1.34 | 0 (0-19) |
| Prior tetracyclines | 5179 | 2838 | 0.73 | 1.37 | 0 (0-20) |
| Prior penicillins | 5390 | 2971 | 0.76 | 1.44 | 0 (0-38) |
| Prior sulfonamides and trimethoprim | 1581 | 863 | 0.22 | 0.97 | 0 (0-27) |
| Prior MLSBs | 4620 | 2430 | 0.65 | 1.34 | 0 (0-20) |
| Prior quinolones | 1063 | 637 | 0.15 | 0.73 | 0 (0-26) |
| Prior other antibacterials (J01X) | 154 | 87 | 0.020 | 0.27 | 0 (0-9) |

**Supplementary Table 2: Regional variation in ARG load.** For each of the six geographical regions, we show the sample size (N), median ARG diversity, and median ARG load together with the 5% and 95% quantiles, prevalence of the high ARG load individuals (>458 RPKM; calculated based on the top-10% quantile across all regions combined), ratio between the median ARG load between the given region and Lapland, and ratio between the high ARG prevalence between the indicated region and Lapland. The other regions are compared to Lapland, as this region has the largest sample size, the lowest population density, and the lowest average ARG load.

| **Region** | **N** | **ARG diversity** median (quantiles) | **ARG load (RPKM)** median (quantiles) | **High ARG Prevalence** (%) | **Median ARG Ratio** | **High ARG Ratio** |
|---|---|---|---|---|---|---|
| Lapland | 1462 | 2.8 (6.58-29.85) | 218 (86-529) | 7.5 | 1 | 1 |
| Karelia | 939 | 2.81 (6.63-29.67) | 228 (93-553) | 8.4 | 1.04 | 1.12 |
| Savonia | 1087 | 2.85 (5.91-30.26) | 231 (101-549) | 8.9 | 1.06 | 1.19 |
| Oulu | 1420 | 2.84 (6.99-29.9) | 228 (87-565) | 10.4 | 1.04 | 1.39 |
| Turku | 934 | 2.89 (7.63-31.71) | 246 (111-593) | 11 | 1.13 | 1.47 |
| Helsinki | 1253 | 2.94 (8.57-34.92) | 263 (110-631) | 13.8 | 1.2 | 1.84 |

**Supplementary Table 3: Regional ARG load variation and demographic factors.** Associations between ARG load (log10 RPKM) and the indicated background factors. Linear models were fitted separately for each sex (2) and geographical sub-region (6). In addition, results are shown for Eastern and Western Finland; Western Finland covers the urban

regions of Helsinki and Turku; Eastern Finland covers the other four regions. The effect size (exponent of the slope fitted on logarithmic ARG load) indicates the relative increase in ARG load per each level of the corresponding variable; the effect size >1 indicates a positive association. Antibiotic use and other covariates have been omitted in these sex- and region-specific models due to low sample sizes and limited power. The analyses for the entire cohort are adjusted for antibiotic consumption and other covariates (Supplementary Data 1 and Fig. 2b).

| Variable | Region | Sex | Effect size | FDR |
|---|---|---|---|---|
| Baseline age | Lapland | Women | 1.001 | 0.584 |
| Household income level | Lapland | Women | 1.033 | **0.006** |
| Raw vegetables and salad | Lapland | Women | 1.023 | 0.240 |
| Poultry meat | Lapland | Women | 1.029 | 0.240 |
| Population density (log10) | Lapland | Women | 1.019 | 0.492 |
| Baseline age | Lapland | Men | 0.995 | **0.018** |
| Household income level | Lapland | Men | 1.042 | **0.003** |
| Raw vegetables and salad | Lapland | Men | 1.04 | **0.05** |
| Poultry meat | Lapland | Men | 1.045 | 0.066 |
| Population density (log10) | Lapland | Men | 1.055 | **0.05** |
| Baseline age | Oulu | Women | 1 | 0.926 |
| Household income level | Oulu | Women | 1.005 | 0.812 |
| Raw vegetables and salad | Oulu | Women | 1.027 | 0.434 |
| Poultry meat | Oulu | Women | 1.018 | 0.632 |
| Population density (log10) | Oulu | Women | 1.03 | 0.434 |
| Baseline age | Oulu | Men | 0.998 | 0.305 |
| Household income level | Oulu | Men | 1.031 | **0.037** |
| Raw vegetables and salad | Oulu | Men | 1.032 | 0.138 |
| Poultry meat | Oulu | Men | 1.053 | 0.064 |
| Population density (log10) | Oulu | Men | 1.025 | 0.305 |
| Baseline age | Helsinki | Women | 1.002 | 0.33 |
| Household income level | Helsinki | Women | 0.998 | 0.798 |
| Raw vegetables and salad | Helsinki | Women | 0.979 | 0.432 |
| Poultry meat | Helsinki | Women | 1.014 | 0.627 |
| Population density (log10) | Helsinki | Women | 1.155 | 0.064 |
| Baseline age | Helsinki | Men | 1.002 | 0.247 |
| Household income level | Helsinki | Men | 1.021 | 0.065 |

| | | | | |
|---|---|---|---|---|
| Raw vegetables and salad | Helsinki | Men | 0.994 | 0.725 |
| Poultry meat | Helsinki | Men | 1.059 | 0.054 |
| Population density (log10) | Helsinki | Men | 1.077 | 0.247 |
| Baseline age | Karelia | Women | 1.003 | 0.42 |
| Household income level | Karelia | Women | 1.004 | 0.88 |
| Raw vegetables and salad | Karelia | Women | 1.003 | 0.88 |
| Poultry meat | Karelia | Women | 1.033 | 0.42 |
| Population density (log10) | Karelia | Women | 1.025 | 0.65 |
| Baseline age | Karelia | Men | 0.999 | 0.793 |
| Household income level | Karelia | Men | 1.036 | **0.047** |
| Raw vegetables and salad | Karelia | Men | 1.025 | 0.372 |
| Poultry meat | Karelia | Men | 1.037 | 0.372 |
| Population density (log10) | Karelia | Men | 0.991 | 0.793 |
| Baseline age | Kuopio | Women | 1.003 | 0.222 |
| Household income level | Kuopio | Women | 0.999 | 0.893 |
| Raw vegetables and salad | Kuopio | Women | 1.028 | 0.214 |
| Poultry meat | Kuopio | Women | 1.034 | 0.214 |
| Population density (log10) | Kuopio | Women | 1.049 | 0.164 |
| Baseline age | Kuopio | Men | 1.002 | 0.47 |
| Household income level | Kuopio | Men | 1.02 | 0.36 |
| Raw vegetables and salad | Kuopio | Men | 0.982 | 0.47 |
| Poultry meat | Kuopio | Men | 1.038 | 0.36 |
| Population density (log10) | Kuopio | Men | 1.01 | 0.697 |
| Baseline age | Turku | Women | 1 | 0.921 |
| Household income level | Turku | Women | 0.999 | 0.921 |
| Raw vegetables and salad | Turku | Women | 0.988 | 0.904 |
| Poultry meat | Turku | Women | 0.979 | 0.836 |
| Population density (log10) | Turku | Women | 1.031 | 0.836 |
| Baseline age | Turku | Men | 1.003 | 0.344 |
| Household income level | Turku | Men | 1.014 | 0.344 |
| Raw vegetables and | Turku | Men | 1.021 | 0.344 |

| | | | | |
|---|---|---|---|---|
| salad | | | | |
| Poultry meat | Turku | Men | 1.062 | 0.139 |
| Population density (log10) | Turku | Men | 1.031 | 0.344 |
| Baseline age | East | Women | 1.001 | 0.287 |
| Household income level | East | Women | 1.012 | **0.037** |
| Raw vegetables and salad | East | Women | 1.024 | **0.012** |
| Poultry meat | East | Women | 1.029 | **0.012** |
| Population density (log10) | East | Women | 1.032 | **0.012** |
| Baseline age | East | Men | 0.998 | **0.026** |
| Household income level | East | Men | 1.033 | **< 0.001** |
| Raw vegetables and salad | East | Men | 1.024 | **0.019** |
| Poultry meat | East | Men | 1.046 | **0.001** |
| Population density (log10) | East | Men | 1.027 | **0.042** |
| Baseline age | West | Women | 1.002 | 0.201 |
| Household income level | West | Women | 0.999 | 0.888 |
| Raw vegetables and salad | West | Women | 0.984 | 0.388 |
| Poultry meat | West | Women | 1.002 | 0.888 |
| Population density (log10) | West | Women | 1.071 | **0.03** |
| Baseline age | West | Men | 1.003 | **0.069** |
| Household income level | West | Men | 1.019 | **0.027** |
| Raw vegetables and salad | West | Men | 1.005 | 0.698 |
| Poultry meat | West | Men | 1.063 | **0.002** |
| Population density (log10) | West | Men | 1.05 | 0.069 |

**Supplementary Table 4: Dissimilarity in resistome composition between regions.** Dissimilarity between the six geographic regions in Finland (PERMANOVA; Bray-Curtis index, N = 7,095 participants). DF = residual degrees of freedom, F = F statistics. Significant FDR-adjusted P-values are bolded.

| **Pairs** | **DF** | **F** | **R2** | **FDR** |
|---|---|---|---|---|
| Lapland vs Oulu | 2880 | 3.0 | 0.001 | **0.005** |
| Lapland vs Helsinki | 2713 | 26.3 | 0.010 | **0.002** |
| Lapland vs Karelia | 2399 | 2.5 | 0.001 | **0.010** |

| | | | | |
|---|---|---|---|---|
| Lapland vs Kuopio | 2547 | 4.6 | 0.002 | **0.002** |
| Lapland vs Turku | 2394 | 14.0 | 0.006 | **0.002** |
| Oulu vs Helsinki | 2671 | 14.4 | 0.005 | **0.002** |
| Oulu vs Karelia | 2357 | 2.2 | 0.001 | **0.022** |
| Oulu vs Kuopio | 2505 | 1.4 | 0.001 | 0.121 |
| Oulu vs Turku | 2352 | 6.3 | 0.003 | **0.002** |
| Helsinki vs Karelia | 2190 | 14.7 | 0.007 | **0.002** |
| Helsinki vs Kuopio | 2338 | 11.5 | 0.005 | **0.002** |
| Helsinki vs Turku | 2185 | 3.2 | 0.001 | **0.002** |
| Karelia vs Kuopio | 2024 | 1.5 | 0.001 | 0.121 |
| Karelia vs Turku | 1871 | 7.7 | 0.004 | **0.002** |
| Kuopio vs Turku | 2019 | 5.0 | 0.002 | **0.002** |
| Eastern vs Western | 7093 | 29.5 | 0.004 | **0.001** |

**Supplementary Table 5: Associations between enterosignatures and ARG class abundance.** Associations (Figure 3) between the total (summed) abundance of antimicrobial gene classes as detected by ResFinder and each enterosignature (two-sided Kendall's rank correlation (Tau); FDR correction; no covariates, N = 7,095 participants). The ARG load, ARG diversity, and species diversity are also shown. ES-Bact is characterized by *Bacteroides*, ES-Firm by *Firmicutes*, ES-Prev by *Prevotella*, ES-Bifi by *Bifidobacteria*, ES- Esch by *Escheria*. Significant FDR-adjusted P-values are bolded.

| **Variable** | **Enterosignature** | **Tau** | **FDR** |
|---|---|---|---|
| Aminoglycoside | ES-Bact | -0.05 | 2.46E-09 |
| Amphenicol | ES-Bact | -0.02 | 0.022 |
| Beta-lactam | ES-Bact | 0 | 0.620 |
| MLSB | ES-Bact | 0.09 | <1E-16 |
| Tetracycline | ES-Bact | 0.05 | 3.35E-10 |
| Aminoglycoside | ES-Firm | 0.08 | <1E-16 |
| Amphenicol | ES-Firm | 0.03 | 0.00022 |
| Beta-lactam | ES-Firm | -0.02 | 0.0048 |
| MLSB | ES-Firm | 0.06 | 3.91042E-11 |
| Tetracycline | ES-Firm | 0.04 | 2.43E-08 |
| Aminoglycoside | ES-Prev | -0.08 | <1E-16 |
| Amphenicol | ES-Prev | -0.04 | 9.40E-05 |

| | | | |
|---|---|---|---|
| Beta-lactam | ES-Prev | 0.12 | <1E-16 |
| MLSB | ES-Prev | -0.16 | <1E-16 |
| Tetracycline | ES-Prev | -0.1 | <1E-16 |
| Aminoglycoside | ES-Bifi | 0.04 | 1.66E-06 |
| Amphenicol | ES-Bifi | 0.02 | 0.0059 |
| Beta-lactam | ES-Bifi | -0.07 | <1E-16 |
| MLSB | ES-Bifi | 0.04 | 2.08E-06 |
| Tetracycline | ES-Bifi | 0.07 | 2E−16 |
| Aminoglycoside | ES-Esch | 0.21 | <1E-16 |
| Amphenicol | ES-Esch | 0.1 | <1E-16 |
| Beta-lactam | ES-Esch | 0.03 | 0.0042 |
| MLSB | ES-Esch | 0.1 | <1E-16 |
| Tetracycline | ES-Esch | 0.14 | <1E-16 |
| ARG load | ES-Bact | 0.1 | <1E-16 |
| ARG diversity | ES-Bact | -0.1 | <1E-16 |
| Species diversity | ES-Bact | -0.04 | 6.87E-08 |
| ARG load | ES-Firm | 0.05 | 1.62E-08 |
| ARG diversity | ES-Firm | 0.06 | 6.3E-15 |
| Species diversity | ES-Firm | 0.21 | <1E-16 |
| ARG load | ES-Prev | -0.11 | <1E-16 |
| ARG diversity | ES-Prev | -0.14 | <1E-16 |
| Species diversity | ES-Prev | -0.07 | 1.8E-15 |
| ARG load | ES-Bifi | -0.01 | 0.38 |
| ARG diversity | ES-Bifi | 0.14 | <1E-16 |
| Species diversity | ES-Bifi | 0.19 | <1E-16 |
| ARG load | ES-Esch | 0.08 | <1E-16 |
| ARG diversity | ES-Esch | 0.18 | <1E-16 |
| Species diversity | ES-Esch | 0.07 | <1E-16 |

**Supplementary Table 6: Proportional hazards for total mortality in a 17-year follow-up.** Median effect size per each unit increase of the indicated variable and the 95% credible intervals for the probabilistic multivariate Cox proportional hazards model (see Methods, N = 7,095 participants).

| Variable | Median HR | Quantile 2.5% | Quantile 97.5% |
|---|---|---|---|
| Age (at baseline, by 10 years) | 2.56 | 2.40 | 2.74 |
| Current smoker | 2.41 | 2.11 | 2.72 |
| Baseline use, ATC drug class L | 2.27 | 1.56 | 3.22 |
| Men | 1.94 | 1.72 | 2.19 |
| Prevalent diabetes | 1.73 | 1.47 | 2.02 |
| ARG load (log10 RPKM) | 1.34 | 1.07 | 1.67 |
| Baseline blood pressure medication | 1.17 | 1.03 | 1.32 |
| Enterobacteriaceae (log10 rel. abundance) | 1.08 | 1.04 | 1.13 |
| Systolic blood pressure (by 10 mmHg) | 1.04 | 1.01 | 1.07 |
| Raw vegetables and salad | 0.90 | 0.86 | 0.94 |
| Household income level | 0.89 | 0.86 | 0.92 |

**Supplementary Table 7: Proportional hazards for cause-specific mortality in a 17-year follow-up.** Median effect size per each unit increase in ARG load (log10 RPKM) and the 95% credible intervals for the probabilistic multivariate Cox proportional hazards model (see Methods, N = 7,095 participants).

| Cause | Median HR | Quantile 2.5% | Quantile 97.5% | Events (N) |
|---|---|---|---|---|
| Gastrointestinal | 2.72 | 0.95 | 8.28 | 42 |
| Respiratory | 2.52 | 1.29 | 5.08 | 103 |
| Cancer | 1.46 | 0.92 | 2.34 | 221 |
| Cardiovascular | 1.45 | 0.95 | 2.28 | 251 |
| Neurological | 1.41 | 0.59 | 3.64 | 58 |
| All | 1.34 | 1.07 | 1.68 | 947 |
| Physical trauma | 1.13 | 0.49 | 2.69 | 64 |

**Supplementary Table 8: Proportional hazards for incident sepsis in a 17-year follow-up.** Median effect size per each unit increase of the indicated variable and the 95% credible intervals for the probabilistic multivariate Cox proportional hazards model (see Methods, N = 7,095 participants).

| Variable | Median HR | Quantile 2.5% | Quantile 97.5% |
|---|---|---|---|
| Baseline use of ATC drug class L | 2.90 | 1.33 | 5.54 |
| ARG load (log10 RPKM) | 2.22 | 1.33 | 3.65 |
| Age (at baseline, by 10 years) | 1.99 | 1.73 | 2.29 |
| Prevalent diabetes | 1.99 | 1.45 | 2.70 |
| Baseline blood pressure medication | 1.64 | 1.27 | 2.09 |
| Current smoker | 1.63 | 1.21 | 2.19 |
| Men | 1.50 | 1.16 | 1.92 |

| | | | |
|---|---|---|---|
| BMI | 1.04 | 1.01 | 1.06 |
| Household income level | 0.91 | 0.85 | 0.97 |

**Supplementary Table 9: Variable correlations**. Two-sided Pearson correlation (r) and the corresponding FDR-corrected P-values (in parentheses) and participants (N) for pairwise comparisons between the covariates in Fig. 2; calculated using the *rcorr* function from the *Hmisc* R package. See Methods for more details on the covariates levels.

| | **Population density (log10)** | **Raw vegetables and salad** | **Household income level** | **Baseline age** | **Prior antibiotics events** | **BMI** | **Cholesterol** |
|---|---|---|---|---|---|---|---|
| Population density (log10) | 1.00 (NA) 6965 | 0.12 (<1E-16) 6923 | 0.06 (1.0E-07) 6791 | -0.04 (0.002) 6965 | 0.02 (0.115) 6965 | -0.09 (9.5E-13) 6963 | -0.07 (1.6E-08) 6965 |
| Raw vegetables and salad | 0.12 (<1E-16) 6923 | 1.00 (NA) 7051 | 0.24 (<1E-16) 6884 | -0.01 (0.607) 7051 | 0.03 (0.007) 7051 | -0.08 (2.4E-11) 7049 | -0.06 (2.5E-07) 7051 |
| Household income level | 0.06 (< 1.E-07) 6791 | 0.24 (<1E-16) 6884 | 1.00 (NA) 6913 | -0.15 (<1E-16) 6913 | -0.01 (0.390) 6913 | -0.10 (<1E-16) 6911 | -0.02 (0.045) 6913 |
| Baseline age | -0.04 (0.002) 6965 | -0.01 (0.607) 7051 | -0.15 (<1E-16) 6913 | 1.00 (NA) 7095 | 0.03 (0.006) 7095 | 0.26 (<1E-16) 7093 | 0.25 (<1E-16) 7095 |
| Prior antibiotics events | 0.02 (0.115) 6965 | 0.03 (0.007) 7051 | -0.01 (0.390) 6913 | 0.03 (0.006) 7095 | 1.00 (NA) 7095 | 0.08 (6.0E-12) 7093 | -0.06 (3.38E-06) 7095 |
| BMI | -0.09 (9.5E-13) 6963 | -0.08 (2.4E-11) 7049 | -0.10 (<1E-16) 6911 | 0.26 (<1E-16) 7093 | 0.08 (6.0E-12) 7093 | 1.00 (NA) 7093 | 0.15 (<1E-16) 7095 |
| Cholesterol | -0.07 (1.6E-08) 6963 | -0.06 (2.5E-11) 7051 | -0.02 (0.045) 6913 | 0.25 (<1E-16) 7095 | -0.06 (3.4E-06) 7095 | 0.15 (<1E-16) 7093 | 1.00 (NA) 7095 |

**Supplementary Table 10: Association between ARG load and bacterial families.** The associations were quantified by predicting ARG load based on the log10 relative abundances of the prevalent bacterial families and other covariates (boosted GLM, two-sided, N = 7,095, 70/30 train/test split, 4397 degrees of freedom). The estimated effect size indicates the increase in log10 ARG load per unit change, controlled for the other covariates. The 95% confidence interval is shown by lower (2.5%) and upper (97.5%) limits and the (unadjusted) P-value. The bacterial families were identified with MetaPhlan3. A complementary analysis with MetaPhlAn4 is provided in Supplementary Table 11.

| **Variable** | **Est.** | **2.50%** | **97.50%** | **exp Est.** | **exp 2.5%** | **exp 97.5%** | **% change** | **P** |
|---|---|---|---|---|---|---|---|---|
| Bacteroidaceae | 0.019 | 0.01 | 0.03 | 1.046 | 1.02 | 1.072 | 4.589 | 3.93E-04 |
| Bifidobacteriaceae | -0.024 | -0.03 | -0.018 | 0.946 | 0.933 | 0.959 | -5.399 | 1.12E-15 |
| Baseline age | -0.001 | 0 | 0.001 | 0.998 | 0.997 | 0.999 | -0.213 | 3.84E-04 |
| Baseline use ATC M01 | 0.008 | -0.01 | 0.031 | 1.019 | 0.967 | 1.073 | 1.861 | 4.89E-01 |
| Baseline use J | 0.01 | -0.02 | 0.037 | 1.022 | 0.96 | 1.089 | 2.233 | 4.93E-01 |
| Baseline use antibiotics | 0.065 | 0.04 | 0.091 | 1.162 | 1.093 | 1.234 | 16.162 | 1.31E-06 |
| Baseline use MLSBs | 0.017 | -0.02 | 0.049 | 1.039 | 0.965 | 1.12 | 3.948 | 3.06E-01 |
| Clostridiales_unclas | 0.004 | 0 | 0.009 | 1.009 | 0.997 | 1.021 | 0.907 | 1.47E-01 |

| | | | | | | | | |
|---|---|---|---|---|---|---|---|---|
| sified | | | | | | | | |
| Coriobacteriaceae | -0.005 | -0.01 | 0.001 | 0.988 | 0.976 | 1 | -1.181 | 5.97E-02 |
| Eastern Finland | -0.014 | -0.03 | 0.001 | 0.968 | 0.935 | 1.001 | -3.223 | 6.01E-02 |
| Enterobacteriaceae (log10 rel. abundance) | 0.017 | 0.01 | 0.021 | 1.04 | 1.029 | 1.051 | 3.959 | 8.35E-13 |
| Eubacteriaceae | 0.004 | 0 | 0.011 | 1.01 | 0.995 | 1.026 | 1.008 | 2.04E-01 |
| Firmicutes_unclassified | 0.004 | 0 | 0.009 | 1.009 | 0.997 | 1.02 | 0.877 | 1.31E-01 |
| Cholesterol | -0.001 | -0.01 | 0.004 | 0.997 | 0.984 | 1.01 | -0.298 | 6.49E-01 |
| Raw vegetables and salad | 0.002 | 0 | 0.007 | 1.006 | 0.994 | 1.017 | 0.563 | 3.34E-01 |
| Poultry meat | 0.006 | 0 | 0.012 | 1.013 | 1 | 1.028 | 1.344 | 5.78E-02 |
| Men | -0.018 | -0.03 | -0.006 | 0.958 | 0.932 | 0.986 | -4.152 | 3.15E-03 |
| Oscillospiraceae | 0.003 | 0 | 0.008 | 1.008 | 0.997 | 1.019 | 0.766 | 1.74E-01 |
| Prior ATC A | 0.023 | 0.01 | 0.038 | 1.055 | 1.02 | 1.09 | 5.456 | 1.75E-03 |
| Prior ATC A07 events | 0.003 | 0 | 0.005 | 1.006 | 1.001 | 1.012 | 0.636 | 1.54E-02 |
| Prior ATC ATC M01 events | 0.001 | 0 | 0.002 | 1.001 | 0.998 | 1.004 | 0.116 | 4.35E-01 |
| Prior ATC B | 0.012 | -0.01 | 0.037 | 1.027 | 0.969 | 1.089 | 2.705 | 3.71E-01 |
| Prior ATC D events | 0.002 | 0 | 0.004 | 1.005 | 1.001 | 1.009 | 0.479 | 1.45E-02 |
| Prior ATC J | 0.049 | 0.03 | 0.066 | 1.12 | 1.078 | 1.164 | 12.033 | 8.14E-09 |
| Prior antibiotics events | -0.002 | 0 | 0.001 | 0.996 | 0.991 | 1.002 | -0.363 | 1.71E-01 |
| Prior tetracyclines | 0.119 | 0.1 | 0.136 | 1.314 | 1.262 | 1.367 | 31.384 | 4.26E-40 |
| Prior tetracyclines events | 0.021 | 0.01 | 0.028 | 1.049 | 1.032 | 1.066 | 4.901 | 3.71E-09 |
| Prior MLSBs | 0.016 | 0 | 0.033 | 1.037 | 0.996 | 1.08 | 3.727 | 7.52E-02 |
| Prior MLSBs events | 0.01 | 0 | 0.017 | 1.023 | 1.007 | 1.04 | 2.348 | 5.59E-03 |
| Prior other antibacterials (J01X) events | -0.017 | -0.04 | 0.003 | 0.963 | 0.92 | 1.007 | -3.747 | 1.01E-01 |
| Prior ATC N05 | 0.015 | 0 | 0.033 | 1.035 | 0.992 | 1.08 | 3.515 | 1.12E-01 |
| Prior ATC R | 0.018 | 0 | 0.031 | 1.043 | 1.011 | 1.075 | 4.259 | 7.91E-03 |
| Prevotellaceae | -0.004 | -0.01 | 0.001 | 0.992 | 0.983 | 1.001 | -0.813 | 6.56E-02 |
| Rikenellaceae | 0.008 | 0 | 0.013 | 1.018 | 1.006 | 1.031 | 1.849 | 2.47E-03 |
| Ruminococcaceae | 0.026 | 0.01 | 0.046 | 1.062 | 1.015 | 1.112 | 6.23 | 9.39E-03 |

| | | | | | | | | |
|---|---|---|---|---|---|---|---|---|
| Bacterial species diversity | -0.001 | -0.02 | 0.019 | 0.999 | 0.955 | 1.044 | -0.13 | 9.55E-01 |
| Tannerellaceae | 0.007 | 0 | 0.013 | 1.017 | 1.004 | 1.029 | 1.658 | 1.02E-02 |
| Household income level | 0.003 | 0 | 0.006 | 1.007 | 1 | 1.014 | 0.707 | 3.61E-02 |
| Library size (million reads) | 0.001 | 0 | 0.001 | 1 | 1 | 1 | 0.001 | 2.62E-01 |
| Population density (log10) | 0.001 | 0 | 0.001 | 1 | 1 | 1 | 0.001 | 1.48E-03 |

**Supplementary Table 11: Association between ARG load and bacterial families.** Reanalysis of the results in Supplementary Table 10 with taxonomic profiles obtained with MetaPhlAn4. The associations were quantified by predicting ARG load based on the log10 relative abundances of the prevalent bacterial families and other covariates (boosted GLM, two-sided, N = 7,095, 70/30 train/test split, 4397 degrees of freedom). The estimated effect size indicates the increase in log10 ARG load per unit change, controlled for the other covariates. The 95% confidence interval is shown by lower (2.5%) and upper (97.5%) limits and the (unadjusted) P-value.

| Variable | Est. | 2.5% | 97.5% | exp Est. | exp 2.5% | exp 97.5% | % change | P |
|---|---|---|---|---|---|---|---|---|
| Baseline age | -0.001 | -0.001 | 0.000 | 0.998 | 0.997 | 0.999 | -0.18 | 1.74E-03 |
| Baseline use J | 0.006 | -0.021 | 0.034 | 1.015 | 0.953 | 1.080 | 1.48 | 6.46E-01 |
| Baseline use antibiotics | 0.068 | 0.042 | 0.094 | 1.168 | 1.100 | 1.241 | 16.84 | 3.95E-07 |
| Baseline use MLSBs | 0.013 | -0.019 | 0.045 | 1.031 | 0.957 | 1.110 | 3.06 | 4.24E-01 |
| Eastern Finland | -0.023 | -0.038 | -0.008 | 0.948 | 0.917 | 0.981 | -5.16 | 2.33E-03 |
| Acholeplasmatales_unclassified | -0.030 | -0.040 | -0.021 | 0.933 | 0.913 | 0.953 | -6.73 | 4.69E-10 |
| Alphaproteobacteria_unclassified | -0.005 | -0.010 | 0.000 | 0.988 | 0.977 | 0.999 | -1.20 | 3.06E-02 |
| Bacteroidaceae | 0.009 | 0.001 | 0.018 | 1.021 | 1.001 | 1.041 | 2.12 | 3.48E-02 |
| Bacteroidales_unclassified | 0.011 | 0.004 | 0.018 | 1.026 | 1.010 | 1.042 | 2.59 | 1.57E-03 |
| Bifidobacteriaceae | -0.021 | -0.026 | -0.016 | 0.953 | 0.942 | 0.965 | -4.69 | 4.99E-15 |
| Candidatus_Gastranaerophilales_unclassified | -0.005 | -0.010 | 0.000 | 0.989 | 0.977 | 1.001 | -1.13 | 6.64E-02 |
| Clostridia_unclassified | 0.014 | 0.006 | 0.022 | 1.032 | 1.014 | 1.051 | 3.23 | 5.44E-04 |

| | | | | | | | | |
|---|---|---|---|---|---|---|---|---|
| Clostridiales_Family_XIII_Incertae _Sedis | -15.656 | -28.086 | -3.227 | 0.0000 | 0.000 | 0.001 | -100.00 | 1.36E-02 |
| Coriobacteriaceae | -0.008 | -0.013 | -0.003 | 0.982 | 0.970 | 0.994 | -1.83 | 3.57E-03 |
| Desulfovibrionaceae | -0.005 | -0.010 | -0.001 | 0.988 | 0.977 | 0.999 | -1.22 | 2.59E-02 |
| Enterobacteriaceae (log10 rel. abundance) | 0.015 | 0.010 | 0.019 | 1.034 | 1.023 | 1.046 | 3.41 | 2.96E-09 |
| Lentisphaeria_unclassified | -0.011 | -0.018 | -0.004 | 0.975 | 0.959 | 0.992 | -2.51 | 3.29E-03 |
| Odoribacteraceae | 0.006 | 0.001 | 0.011 | 1.014 | 1.002 | 1.025 | 1.38 | 2.01E-02 |
| Peptostreptococcaceae | -0.010 | -0.016 | -0.003 | 0.978 | 0.963 | 0.994 | -2.19 | 5.51E-03 |
| Phyllobacteriaceae | -0.007 | -0.019 | 0.004 | 0.983 | 0.958 | 1.009 | -1.71 | 1.89E-01 |
| Pirellulaceae | -0.012 | -0.021 | -0.004 | 0.972 | 0.953 | 0.991 | -2.82 | 4.90E-03 |
| Ruminococcaceae | 0.040 | 0.017 | 0.062 | 1.096 | 1.040 | 1.155 | 9.60 | 5.76E-04 |
| Sutterellaceae | -0.008 | -0.013 | -0.004 | 0.981 | 0.971 | 0.992 | -1.90 | 4.46E-04 |
| Veillonellaceae | -0.002 | -0.005 | 0.002 | 0.996 | 0.988 | 1.004 | -0.43 | 2.94E-01 |
| Victivallaceae | -0.011 | -0.032 | 0.009 | 0.974 | 0.929 | 1.022 | -2.58 | 2.83E-01 |
| Cholesterol | -0.001 | -0.007 | 0.004 | 0.997 | 0.985 | 1.010 | -0.29 | 6.58E-01 |
| Raw vegetables and salad | 0.003 | -0.002 | 0.008 | 1.008 | 0.996 | 1.019 | 0.77 | 1.82E-01 |
| Poultry meat | 0.007 | 0.001 | 0.013 | 1.016 | 1.003 | 1.030 | 1.64 | 1.99E-02 |
| Men | -0.019 | -0.031 | -0.007 | 0.957 | 0.931 | 0.984 | -4.30 | 1.97E-03 |
| Prior ATC A | 0.024 | 0.010 | 0.038 | 1.057 | 1.022 | 1.092 | 5.65 | 1.03E-03 |
| Prior ATC A07 events | 0.003 | 0.001 | 0.005 | 1.007 | 1.002 | 1.012 | 0.73 | 5.00E-03 |
| Prior ATC D events | 0.002 | 0.000 | 0.004 | 1.005 | 1.001 | 1.008 | 0.46 | 1.91E-02 |
| Prior ATC J | 0.046 | 0.030 | 0.063 | 1.113 | 1.071 | 1.156 | 11.28 | 4.60E-08 |
| Prior antibiotics events | -0.001 | -0.004 | 0.002 | 0.998 | 0.992 | 1.004 | -0.21 | 5.03E-01 |
| Prior tetracyclines | 0.116 | 0.098 | 0.133 | 1.305 | 1.254 | 1.358 | 30.51 | 8.71E-39 |
| Prior tetracyclines events | 0.019 | 0.012 | 0.027 | 1.046 | 1.029 | 1.063 | 4.58 | 7.40E-08 |
| Prior MLSBs | 0.019 | 0.002 | 0.036 | 1.045 | 1.004 | 1.087 | 4.49 | 3.03E-02 |

| | | | | | | | | |
|---|---|---|---|---|---|---|---|---|
| Prior MLSBs events | 0.009 | 0.002 | 0.016 | 1.021 | 1.005 | 1.038 | 2.14 | 1.09E-02 |
| Prior quinolones events | -0.006 | -0.015 | 0.003 | 0.986 | 0.966 | 1.006 | -1.42 | 1.77E-01 |
| Prior ATC N05 | 0.016 | -0.002 | 0.034 | 1.038 | 0.995 | 1.082 | 3.79 | 8.21E-02 |
| Prior ATC R | 0.021 | 0.008 | 0.034 | 1.050 | 1.018 | 1.083 | 5.00 | 1.72E-03 |
| Bacterial species diversity | 0.024 | 0.007 | 0.042 | 1.058 | 1.016 | 1.101 | 5.76 | 6.39E-03 |
| Household income level | 0.004 | 0.001 | 0.007 | 1.009 | 1.002 | 1.015 | 0.85 | 1.10E-02 |
| Library size (million reads) | 0.000 | 0.000 | 0.000 | 1.0000 | 1.000 | 1.000 | 0.00 | 3.06E-04 |
| Population density (log10) | 0.000 | 0.000 | 0.000 | 1.0000 | 1.000 | 1.000 | 0.00 | 1.36E-03 |

**Supplementary Table 12: Antibiotic use and antibiotic resistance.** The results of linear regression models examine the association between antibiotic purchases in the years preceding sampling and the corresponding antibiotic class's resistance load. Resistance load was modeled using log10(RPKM) and calculated for each antibiotic by summing the RPKM values of all genes within the specific antibiotic class. Antibiotic use was represented as the unscaled number of purchases. The 95% confidence interval is shown by lower (2.5%) and upper (97.5%) limits for the estimate (Est.) and exponentiated estimate (exp Est.) with the FDR corrected P-value (FDR). N = 7,095 samples, 7,093 degrees of freedom.

| **Antibiotic resistance (RPKM load)** | **Covariate** | **Est.** | **2.50 %** | **97.50 %** | **exp Est.** | **exp 2.5%** | **exp 97.5%** | **% change** | **FDR** |
|---|---|---|---|---|---|---|---|---|---|
| Tetracycline | Number of tetracycline purchases | 0.082 | 0.077 | 0.088 | 1.21 | 1.19 | 1.23 | 20.9 | 4.15E-161 |
| Beta-lactam | Number of penicillin purchases | 0.060 | 0.048 | 0.071 | 1.15 | 1.12 | 1.18 | 14.7 | 8.67E-25 |
| Beta-lactam | Number of puchases of other beta-lactams | 0.060 | 0.047 | 0.072 | 1.15 | 1.12 | 1.18 | 14.7 | 1.41E-21 |
| Sulphonamide | Number of purchases of sulphonamides | 0.006 | 0.001 | 0.012 | 1.01 | 1.00 | 1.03 | 1.4 | 4.38E-02 |
| Macrolide | Number of purchases of Macrolides | 0.198 | 0.187 | 0.209 | 1.58 | 1.54 | 1.62 | 57.7 | 8.64E-245 |

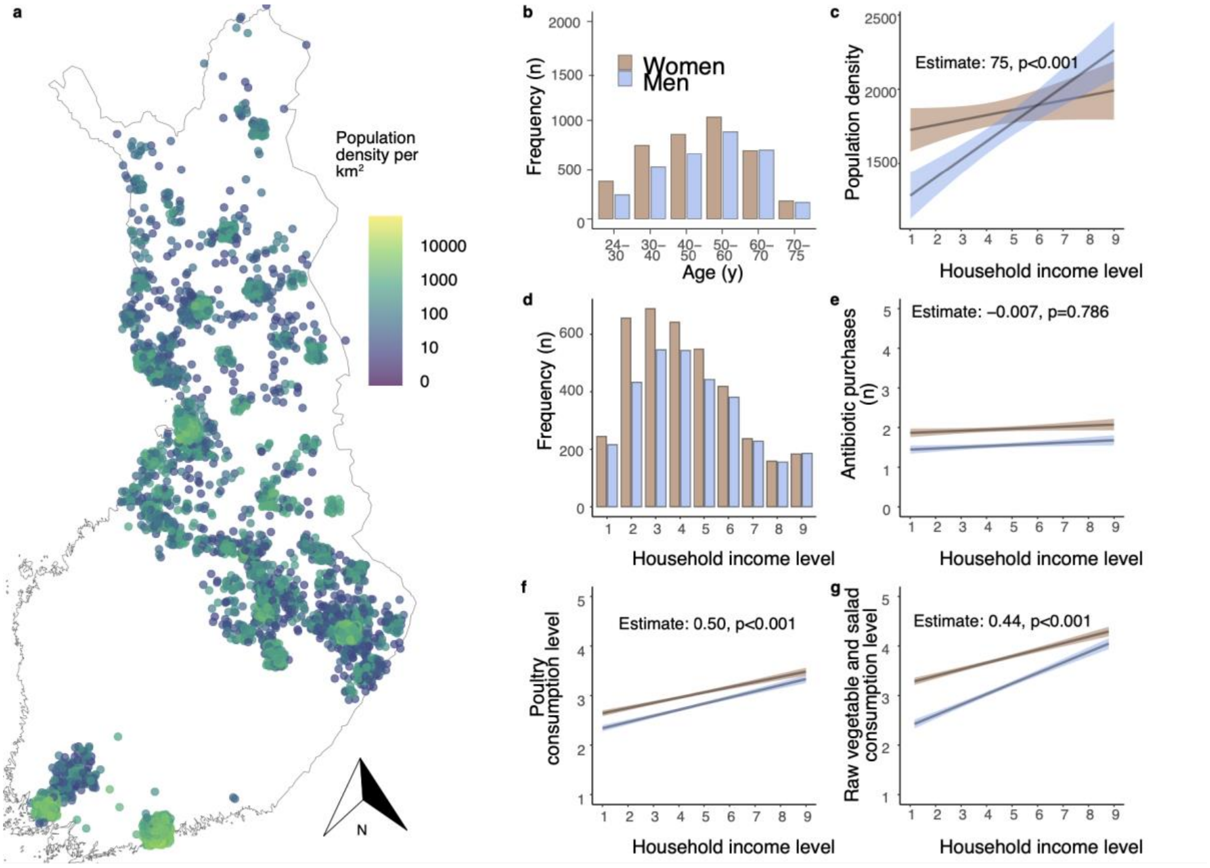


**Supplementary Fig. 1. Overview of the FINRISK cohort (N = 7,095) a** Geographical distribution of the cohort participants. The jittered data points indicate the population density per km2. Some individual points at remote locations were removed to obscure participants' addresses. **b** Age distribution by decade, shown for males (M; blue) and females (F; brown) **c** Population-density versus household income level (scale 1-9). Linear model; two-sided, 6788 degrees of freedom, estimate 75, 95% confidence interval 48 – 102, (unadjusted) P-value < 1 x $10^{-6}$ **d** Household income level (scale 1-9). **e** Household income level versus antibiotic purchases during the seven years before the sample collection. Linear model; 6910 degrees of freedom, estimate -0.007, 95% confidence interval -0.06 - 0.04, unadjusted P-value = 0.786, **f** Household income level versus poultry consumption (scale 1-5, from less than once a month to multiple times per day), Linear model; 6742 degrees of freedom, estimate 0.50, 95% confidence interval 0.45 - 0.55, unadjusted P-value < 1 x $10^{-6}$ and **g** Raw vegetable and salad consumption. Linear model; 6881 degrees of freedom, estimate 0.44, 95% confidence interval 0.40 - 0.48, (unadjusted) P-value < 1 x $10^{-6}$ Panels c, e, f, and g include linear models' estimated slope and unadjusted P-value. See Methods for a more detailed description of the scales.

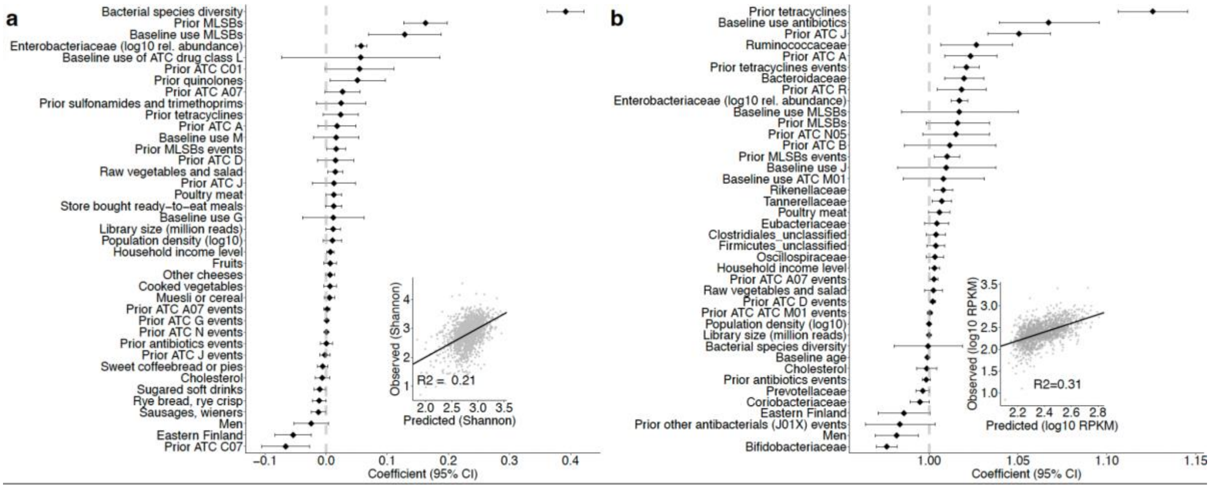


**Supplementary Fig. 2: Drivers of ARG diversity and ARG load a** Drivers of ARG diversity (boosted GLM for ARG Shannon diversity, load (N = 7,095 participants, train/test split 70%/30%, 4397 degrees of freedom). The line plot shows the estimated effect sizes (points) and 95% confidence intervals (bars) for the predictor variables. Inset: Predicted and observed ARG diversity in the leave-out test data. **b** Drivers of ARG load, including bacterial families (MetaPhlAn3), exponentiated estimate shown (boosted GLM for log10 ARG load). Bacterial abundances are indicated as log10 relative abundance. Inset: Predicted and observed ARG load in the leave-out test data. See also Supplementary Table 10 for the corresponding Table and Supplementary Table 11 for the boosted GLM result Table with MetaPhlAn4 bacterial families.

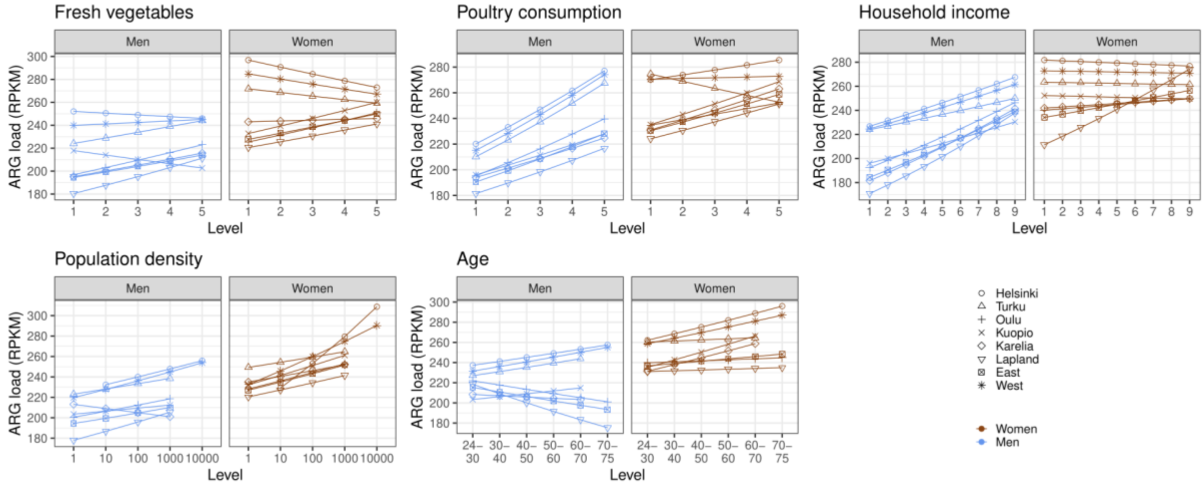


**Supplementary Fig. 3: Regional trends between ARG load and raw vegetables and poultry consumption, household income, population density, and age group.** The figures show the linear model fit between the ARG load (log10 RPKM) and the indicated variable levels (N = 7,095 participants). Separate models for each region and sex were fitted using the R *lm* function. Western Finland covers the urban regions of Helsinki and Turku; Eastern Finland covers the other four regions. The effect sizes (slope) and significance estimates are shown in Supplementary Table 3.

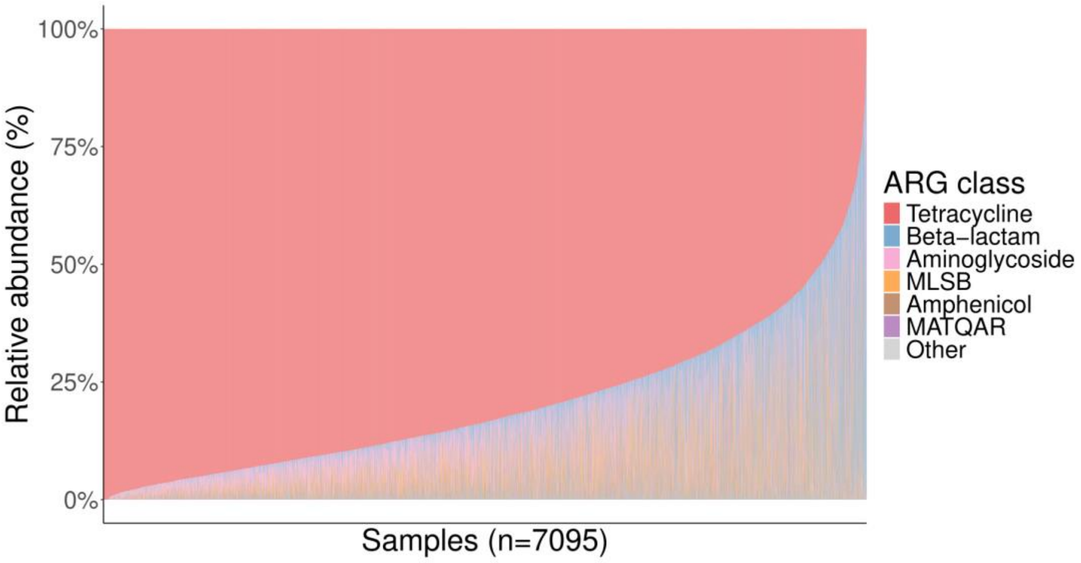


**Supplementary Fig. 4. Relative abundances of the antibiotic resistance gene classes in the FINRISK cohort (N = 7,095).** The classes are defined by the antibiotic class for which the genes confer resistance to (ResFinder). Abbreviations: MLSB: “Macrolide, Lincosamide, Streptogramin B”; MATQAR: "Macrolide, Aminoglycoside, Tetracycline, Quinolone, Amphenicol, Rifamycin".

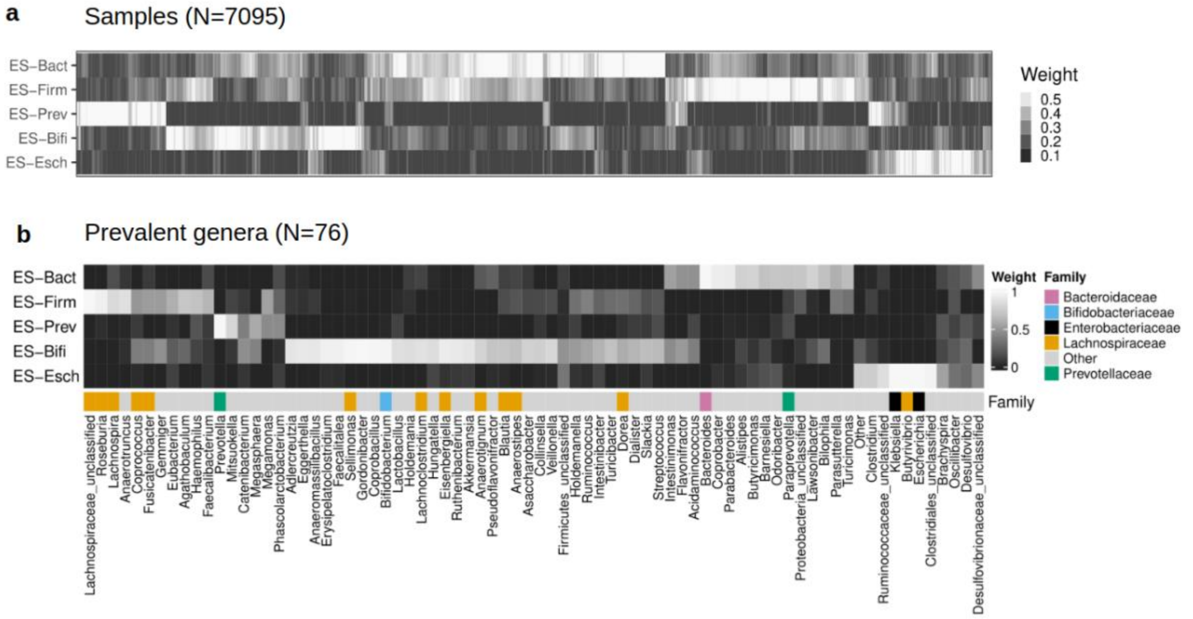


**Supplementary Fig. 5. Enterosignature profiles in FINRISK. a** Sample weights for each enterosignature (ES) across the 7,095 FINRISK participants. The ES scores were normalized by the total sum score per sample. Values above the 95% quantile have been capped for the heatmap visualization. Each individual carries a unique mixture of enterosignatures. **b** Each of the five enterosignatures (ES) represents a mixture of prevalent genera; the heatmap indicates the relative weights for the prevalent genera in each ES (see Methods); the bottom panel indicates their corresponding bacterial families (colors). Associations between each ES and species diversity, ARG diversity, and the total ARG load are provided in Supplementary Table 5. Associations between ES and the resistome: see Supplementary Fig. 6.

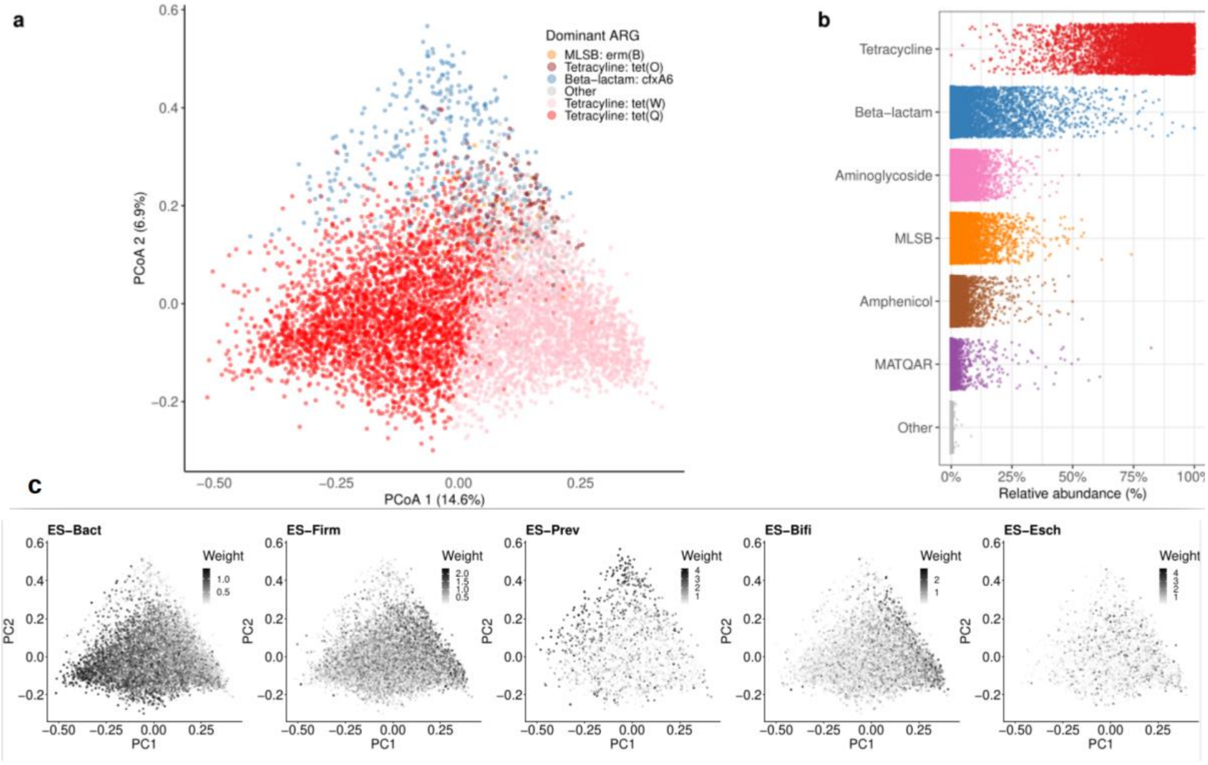

**Supplementary Fig. 6. Resistome landscape. a** Population landscape of human gut resistome composition (PcoA; Bray-Curtis index, N = 7,095 participants). The dominant (most abundant) antibiotic resistance gene for each sample is highlighted by color. **b** Relative abundance distribution for the most abundant ARG classes among the study population (N = 7,095; see also Supplementary Fig. 4). **c** Resistome population variation landscape is linked to bacterial abundance variation. The ordination shows enterosignature weights across the resistome landscape (the normalized NMF score; see Methods). Each enterosignature corresponds to a particular set of co-abundant bacterial genera (Supplementary Fig. 5), and a higher weight indicates a stronger presence of the indicated enterosignature. See Supplementary Data 1 for associations between the dominant bacterial families and ARG load.

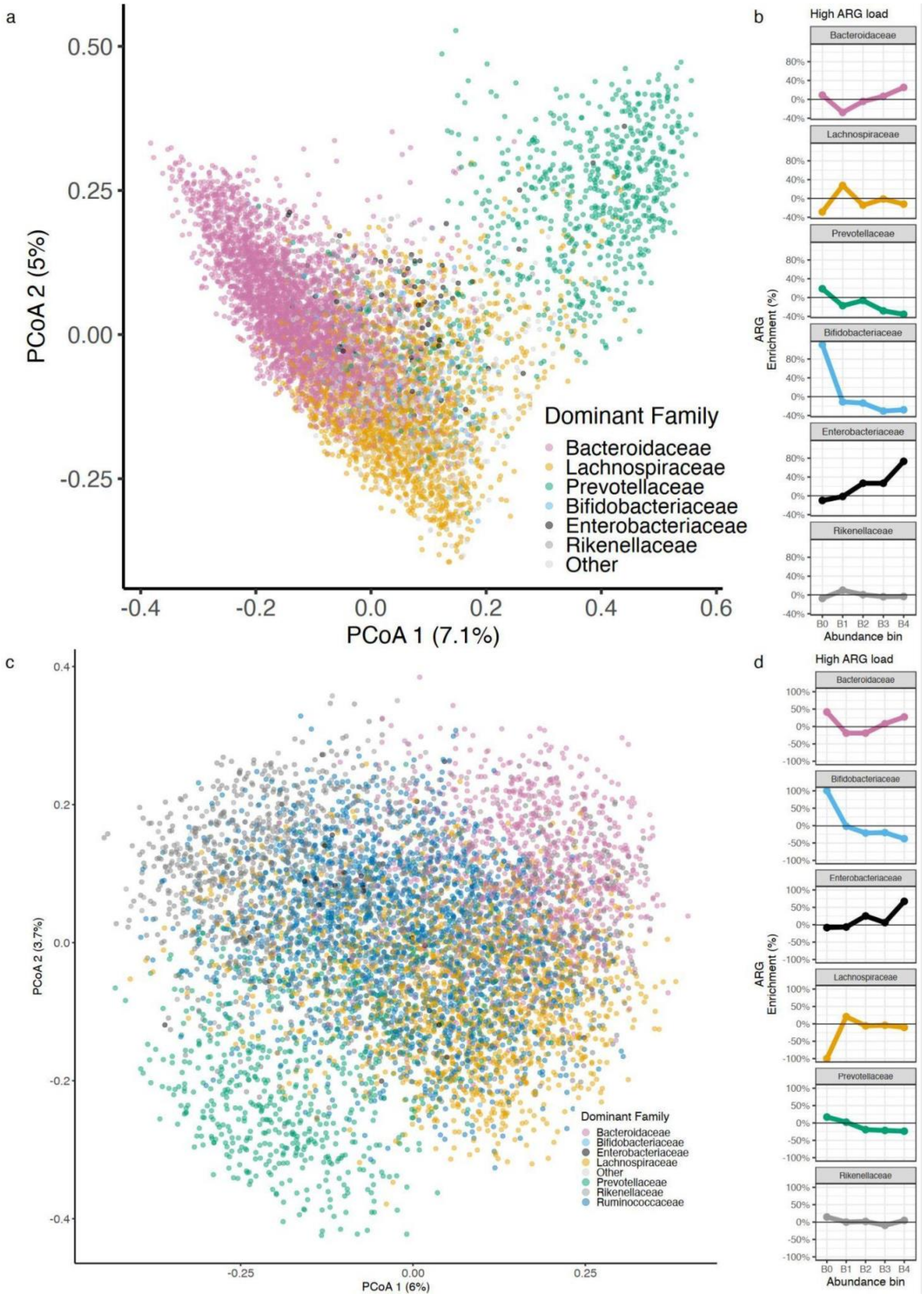


**Supplementary Fig. 7. Population landscape of microbial community composition and observed ARG load. a** Population variation, or landscape, of the microbiota composition among the 7,095 study participants (MetaPhlAn3 species-level PCoA; Bray-Curtis index). Each sample is colored by its most dominant bacterial family. Families that are significantly associated with ARG load and dominant in more than 50 samples are highlighted (Supplementary Data 1; P < 0.05). **b**

The enrichment of individuals with high ARG load (top-10% quantile; >458 RPKM) across the abundance quantiles of each bacterial family in MetaPhlAn3 (B0: not detected; B1-B4 25% abundance quartiles among individuals with detected signal). The estimated prevalence (point) and 95% credible intervals (bars) for the high-ARG individuals are shown within each abundance bin based on a probabilistic Bernoulli model (see Methods). The dashed line indicates the expected prevalence of the high-ARG individuals in the entire study population (10%). **c** PCoA ordination (MetaPhlAn4 species-level PCoA; Bray-Curtis index). The same families are highlighted as in Fig S7a-b. **d** The enrichment of individuals with high ARG load across the abundance quantiles of each bacterial family in MetaPhlAn4. The estimated prevalence (point) and 95% credible intervals (bars) for the high-ARG individuals are shown within each abundance bin based on a probabilistic Bernoulli model (see Methods). The same families are highlighted as in Fig S7a-b.

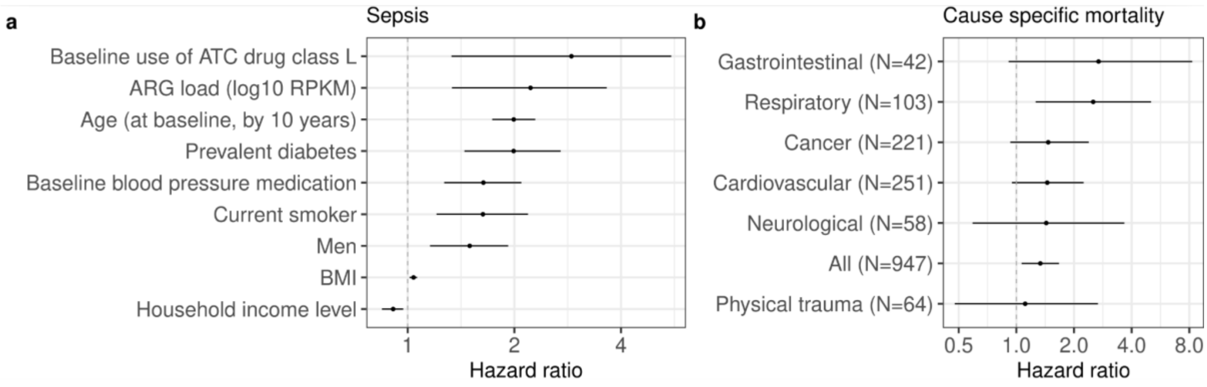


**Supplementary Fig. 8. Factors contributing to sepsis and cause-specific mortality events. a** ARG load is associated with sepsis (N = 6,849, probabilistic multivariate Cox proportional hazards; Supplementary Table 8). The association is robust to adjustment for key covariates. The median hazard ratio (HR) is shown (points) corresponding to a unit increase per each indicated variable, along with the 95% credible intervals (bars). Variables whose credible overlaps with 1 (no association) are excluded from the graph. **b** Associations between ARG load (log10 RPKM) and cause-specific mortality (Supplementary Table 7). The median hazard ratio (HR) is shown corresponding to a unit increase in ARG load (log10 RPKM), along with the 95% credible intervals. Both models were adjusted for age, smoking, sex, diabetes, use of antineoplastic and immunomodulating agents, body mass index, self-reported antihypertensive medication, systolic blood pressure, prior antibiotics use (during the six months before baseline), household income, and raw vegetables and salad consumption (see Methods).

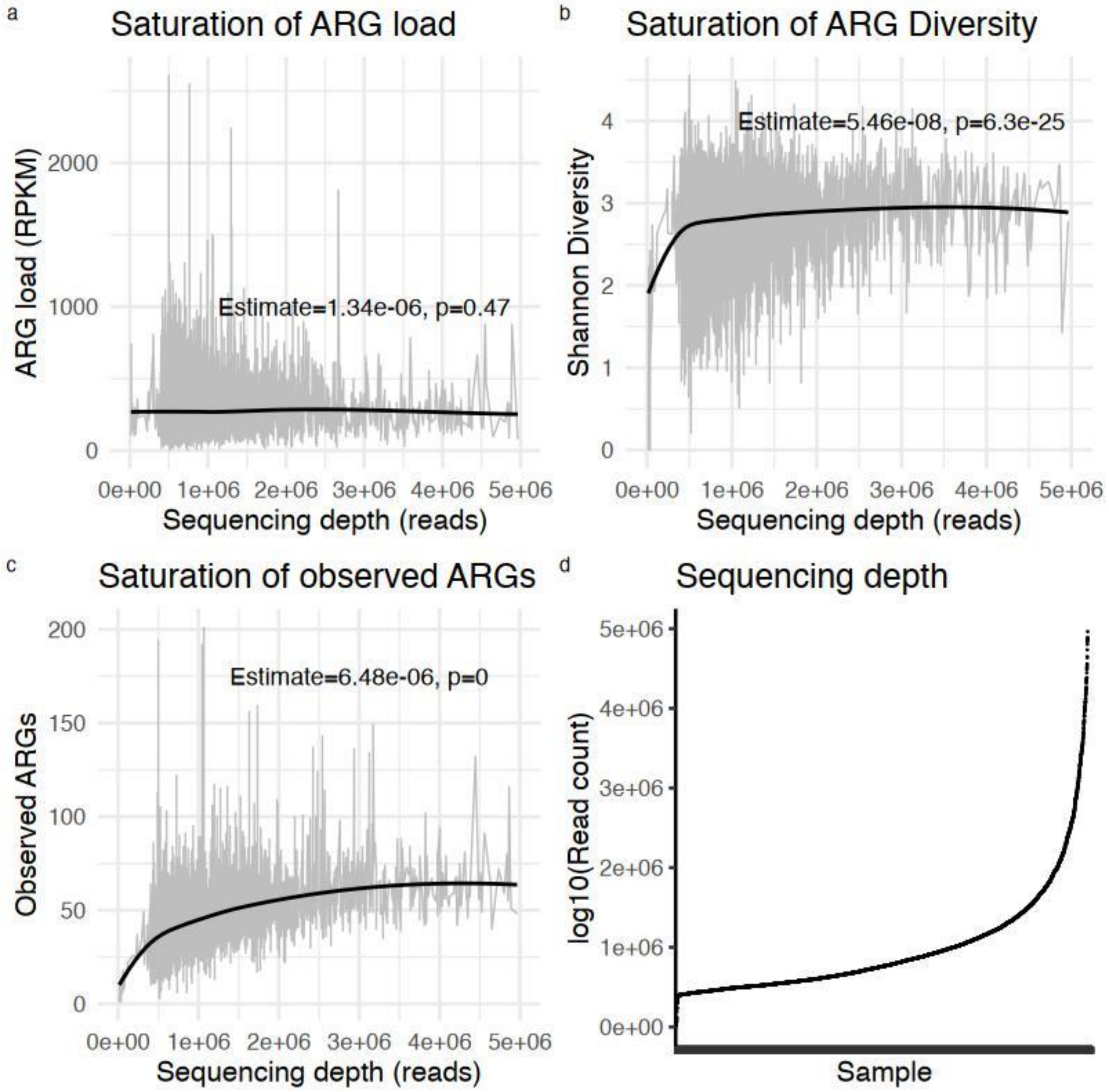


**Supplementary Fig. 9 ARG metrics and sequencing depth a** ARG load in RPKM, **b** ARG Shannon diversity, and **c** observed number of unique ARGs. The y-axes display the respective ARG metrics, while the x-axes show the sequencing depth (the number of reads, N = 7,095 participants). For **a** and **b** linear model effect sizes and the P-values for the respective metric are shown in the figure. The fitted Loess smoothed line is shown in black, while the grey lines depict individual ARG metrics for the corresponding sequencing depth values among the samples (x-axis). **d** sequencing depths of the samples. Each sample is depicted as a point. Samples are arranged along the x-axis in order of increasing sequencing depth. The y-axis shows sequencing depth in log10(number of reads). For panel **c** the P-value is smaller than the threshold for reporting in R (< 2.2E-308).

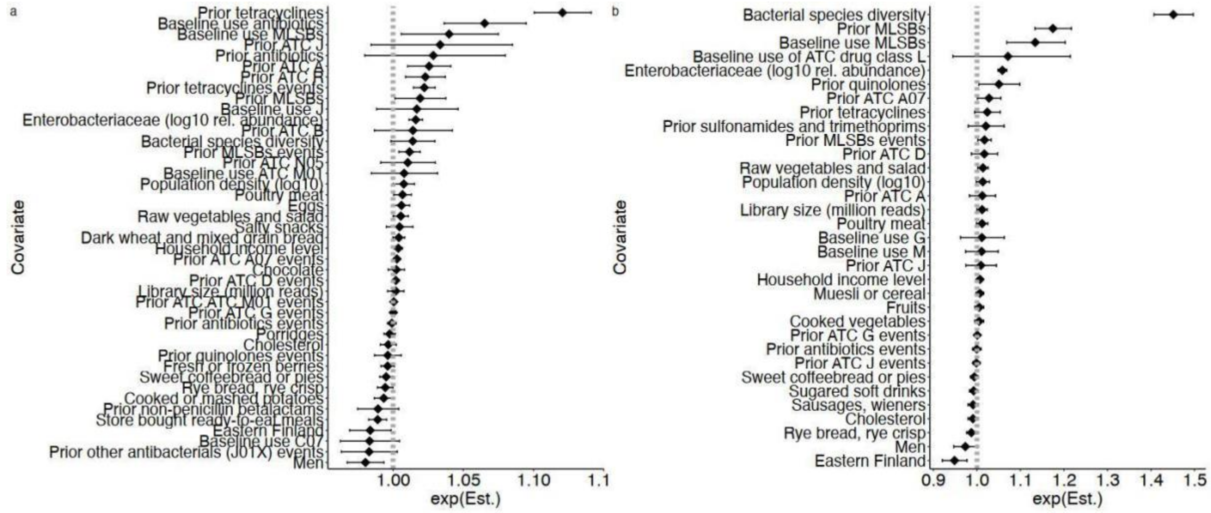


**Supplementary Fig. 10 Drivers of ARG diversity and ARG load in a subset of samples with more than 200,000 reads.** Drivers of ARG load (generalized linear model, GLM for log10 ARG load; N = 7,074 participants, 70%/30% train/ test split, 4463 degrees of freedom). The line plot shows the predictor variables' estimated effect sizes (points), along with their 95% confidence intervals (bars).) **b** Drivers of ARG Shannon diversity (generalized linear model, GLM for log10 ARG load; N = 7,074 participants, 70%/30% train/ test split, 4486 degrees of freedom). Bacterial abundances are indicated as log10 relative abundance. The line plot shows the predictor variables' estimated effect sizes (points), along with their 95% confidence intervals (bars). For panels **a** and **b**, the covariates were selected based on the boosted GLM analysis (See Figure 2). The panels show the estimates for the subset of samples with more than 200,000 reads. The covariates are ordered based on their estimates. The order of the covariates and the magnitude of the estimates are the same as in Figure 2, confirming that removing low-sequencing-depth samples does not qualitatively change the results.
