# Supplementary material for: Variation and prognostic potential of the gut antibiotic resistome in the FINRISK 2002 cohort
Source: Nat Commun. 2025 Jul 1;16:5963. doi: 10.1038/s41467-025-61137-x (PMC12214822; doi:10.1038/s41467-025-61137-x)
Supplement: Supplementary file 2 — Description of Additional Supplementary Files [file 41467_2025_61137_MOESM2_ESM.docx]

Description of Additional Supplementary Files

**File Name:** Supplementary Data 1

**Description:** Linear models with ARG load/diversity and participant level data or bacterial families ARG load, controlled: Associations between ARG load and participant covariates, controlled for prior antibiotic use ARG load, not controlled: Associations between ARG load and participant covariates, not controlled for prior antibiotic use ARG diversity, not controlled: Associations between ARG diversity and participant covariates, not controlled for antibiotic use Bacterial family and ARG load: Association between ARG load, and species and ARG diversity and prevalent microbial families using Kendall's tau; FDR correction; no covariates Women, ARG load, controlled for AB: Associations between ARG load and participant covariates in women, controlled for prior antibiotic use Women, ARG load, not controlled: Associations between ARG load and participant covariates in women, not controlled for prior antibiotic use.

**File Name:** Supplementary Data 2

**Description:** Associations of diet variables with bacterial families and enterosignatures Families: Linear model results for bacterial families relative abundances and diet associations, FDRadjusted p-values. Only significant associations are shown Enterosignatures: Linear model results for enterosignatures and diet associations, FDRadjusted p-values. Only significant associations are shown Correlation matrix: Pearson correlations of diet variables with each other.

**File Name:** Supplementary Data 3

**Description:** Estimated mean and 90% credible intervals for key covariates Fresh vegetable consumption: Summary statistics for fresh vegetable consumption Poultry consumption: Summary statistics for poultry consumption Household income: Summary statistics for household income Population density: Summary statistics for population density Age group: Statistics for Age group.

**File Name:** Supplementary Data 4

**Description:** MetaPhlAn and ARG mapping results from negative controls MetaPhlAn taxa in neg. controls: Contains MetaPlAn3 outputs from the negative controls sequenced with the samples. Columns are names of controls, taxa column has the identified taxa ARGs in neg. controls: Contains ARG mapping outputs from the negative controls. Gene_accession_no has the Resfinder ARG variant name, which includes the name of the gene and the NCBI accession from which it was retrieved.
