# Supplementary material for: Variation and prognostic potential of the gut antibiotic resistome in the FINRISK 2002 cohort
Source: Nat Commun. 2025 Jul 1;16:5963. doi: 10.1038/s41467-025-61137-x (PMC12214822; doi:10.1038/s41467-025-61137-x)
Supplement: Supplementary file 7 — Reporting Summary [file 41467_2025_61137_MOESM7_ESM.pdf]

nature portfolio

Corresponding author(s): Katariina Pärnänen, Leo Lahti

Last updated by author(s): May 28, 2025

Reporting Summary

Nature Portfolio wishes to improve the reproducibility of the work that we publish. This form provides structure for consistency and transparency in reporting. For further information on Nature Portfolio policies, see our Editorial Policies and the Editorial Policy Checklist.

Statistics

For all statistical analyses, confirm that the following items are present in the figure legend, table legend, main text, or Methods section.

n/a	Confirmed	
☐	☒	The exact sample size (*n*) for each experimental group/condition, given as a discrete number and unit of measurement
☒	☐	A statement on whether measurements were taken from distinct samples or whether the same sample was measured repeatedly
☐	☒	The statistical test(s) used AND whether they are one- or two-sided<br>*Only common tests should be described solely by name; describe more complex techniques in the Methods section.*
☐	☒	A description of all covariates tested
☐	☒	A description of any assumptions or corrections, such as tests of normality and adjustment for multiple comparisons
☐	☒	A full description of the statistical parameters including central tendency (e.g. means) or other basic estimates (e.g. regression coefficient) AND variation (e.g. standard deviation) or associated estimates of uncertainty (e.g. confidence intervals)
☐	☒	For null hypothesis testing, the test statistic (e.g. *F*, *t*, *r*) with confidence intervals, effect sizes, degrees of freedom and *P* value noted<br>*Give P values as exact values whenever suitable.*
☐	☒	For Bayesian analysis, information on the choice of priors and Markov chain Monte Carlo settings
☒	☐	For hierarchical and complex designs, identification of the appropriate level for tests and full reporting of outcomes
☐	☒	Estimates of effect sizes (e.g. Cohen's *d*, Pearson's *r*), indicating how they were calculated

Our web collection on statistics for biologists contains articles on many of the points above.

Software and code

Policy information about availability of computer code

Data collection	Code was not used to collect data.
Data analysis	All codes are provided in Github with a permanent DOI https://doi.org/10.5281/zenodo.15574151 and have been provided as a zip archive for review purposes.<br>R version 4.3.1 (2023-06-16)<br>Platform: aarch64-apple-darwin20 (64-bit)<br>Running under: macOS Sonoma 14.2.1<br><br>Matrix products: default<br>BLAS: /System/Library/Frameworks/Accelerate.framework/Versions/A/Frameworks/vecLib.framework/Versions/A/libBLAS.dylib<br>LAPACK: /Library/Frameworks/R.framework/Versions/4.3-arm64/Resources/lib/libRlapack.dylib; LAPACK version 3.11.0<br><br>locale:<br>[1] en_US.UTF-8/en_US.UTF-8/en_US.UTF-8/C/en_US.UTF-8/en_US.UTF-8<br><br>time zone: Europe/Helsinki<br>tzcode source: internal<br><br>attached base packages:

```
 [1] parallel  grid      stats4    stats     graphics  grDevices utils     datasets  methods   base

other attached packages:
 [1] broom_1.0.5                 jtools_2.2.2                mgcv_1.9-0
 [4] nlme_3.1-164                pdp_0.8.1                   caret_6.0-94
 [7] lattice_0.21-9              mboost_2.9-8                stabs_0.6-4
[10] Cairo_1.6-2                 brms_2.20.4                 Rcpp_1.0.13
[13] lubridate_1.9.3             forcats_1.0.0               purrr_1.0.2
[16] readr_2.1.4                 tidyr_1.3.1                 tibble_3.2.1
[19] tidyverse_2.0.0             ggtree_3.8.2                ape_5.8
[22] writexl_1.4.2               stringr_1.5.1               ComplexHeatmap_2.16.0
[25] RColorBrewer_1.1-3          dplyr_1.1.4                 reshape2_1.4.4
[28] circlize_0.4.15             data.table_1.16.0           phyloseq_1.44.0
[31] multcomp_1.4-25             TH.data_1.1-2               MASS_7.3-60
[34] survival_3.5-7              mvtnorm_1.3-1               viridis_0.6.5
[37] viridisLite_0.4.2           microViz_0.12.5             patchwork_1.1.3
[40] ggpubr_0.6.0                miaViz_1.8.0                ggraph_2.1.0
[43] ggplot2_3.5.1               mia_1.13.44                 MultiAssayExperiment_1.28.0
[46] cowplot_1.1.1               TreeSummarizedExperiment_2.10.0 Biostrings_2.70.3
[49] XVector_0.42.0              SingleCellExperiment_1.24.0 SummarizedExperiment_1.32.0
[52] Biobase_2.62.0              GenomicRanges_1.54.1        GenomeInfoDb_1.38.8
[55] IRanges_2.36.0              MatrixGenerics_1.14.0       matrixStats_1.4.1
[58] S4Vectors_0.40.2            BiocGenerics_0.48.1

loaded via a namespace (and not attached):
  [1] shinythemes_1.2.0         nnet_7.3-19               DT_0.30
  [4] rstan_2.32.3              vctrs_0.6.5               partykit_1.2-20
  [7] digest_0.6.37             png_0.1-8                 shape_1.4.6
 [10] rbiom_1.0.3               ggrepel_0.9.6             parallelly_1.36.0
 [13] permute_0.9-7             mediation_4.5.0           magick_2.8.2
 [16] reshape_0.8.9             foreach_1.5.2             httpuv_1.6.11
 [19] withr_3.0.1               xfun_0.47                 ggfun_0.1.3
 [22] ellipsis_0.3.2            memoise_2.0.1             ggbeeswarm_0.7.2
 [25] emmeans_1.8.8             gtools_3.9.4              tidytree_0.4.6
 [28] zoo_1.8-12                GlobalOptions_0.1.2       V8_4.4.0
 [31] prettyunits_1.2.0         Formula_1.2-5             promises_1.2.1
 [34] rstatix_0.7.2             globals_0.16.2            rhdf5filters_1.12.1
 [37] ps_1.7.5                  rhdf5_2.44.0              rstudioapi_0.16.0
 [40] miniUI_0.1.1.1            generics_0.1.3            inum_1.0-5
 [43] processx_3.8.2            base64enc_0.1-3           curl_5.1.0
 [46] zlibbioc_1.48.2           ScaledMatrix_1.10.0       polyclip_1.10-6
 [49] quadprog_1.5-8            GenomeInfoDbData_1.2.11   SparseArray_1.2.4
 [52] threejs_0.3.3             xtable_1.8-4              ade4_1.7-22
 [55] doParallel_1.0.17         evaluate_1.0.0            S4Arrays_1.2.1
 [58] hms_1.1.3                 irlba_2.3.5.1             colorspace_2.1-1
 [61] magrittr_2.0.3            later_1.3.1               posterior_1.4.1
 [64] future.apply_1.11.0       DECIPHER_2.30.0           scuttle_1.12.0
 [67] xts_0.13.1                StanHeaders_2.26.28       class_7.3-22
 [70] Hmisc_5.1-3               pillar_1.9.0              iterators_1.0.14
 [73] decontam_1.22.0           compiler_4.3.1            beachmat_2.18.1
 [76] stringi_1.8.4             biomformat_1.28.0         gower_1.0.1
 [79] minqa_1.2.8               plyr_1.8.9                crayon_1.5.3
 [82] abind_1.4-8               scater_1.33.4             gridGraphics_0.5-1
 [85] graphlayouts_1.0.1        bit_4.5.0                 sandwich_3.1-1
 [88] libcoin_1.0-10            shinystan_2.6.0           codetools_0.2-19
 [91] recipes_1.0.8             BiocSingular_1.18.0       crosstalk_1.2.0
 [94] QuickJSR_1.0.7            slam_0.1-53               GetoptLong_1.0.5
 [97] multtest_2.56.0           mime_0.12                 splines_4.3.1
[100] markdown_1.10             sparseMatrixStats_1.14.0  knitr_1.48
[103] blob_1.2.4                utf8_1.2.4                clue_0.3-65
[106] lme4_1.1-35.5             fs_1.6.4                  nnls_1.5
[109] listenv_0.9.0             checkmate_2.3.2           DelayedMatrixStats_1.24.0
[112] pkgbuild_1.4.2            estimability_1.4.1        ggsignif_0.6.4
[115] ggplotify_0.1.2           Matrix_1.6-1.1            callr_3.7.3
[118] tzdb_0.4.0                lpSolve_5.6.21            tweenr_2.0.2
[121] bayesplot_1.10.0          pkgconfig_2.0.3           tools_4.3.1
[124] cachem_1.1.0              RSQLite_2.3.7             DBI_1.2.3
[127] fastmap_1.2.0             rmarkdown_2.28            scales_1.3.0
```

[130] coda_0.19-4	BiocManager_1.30.22	carData_3.0-5
[133] rpart_4.1.21	farver_2.1.2	tidygraph_1.2.3
[136] foreign_0.8-85	cli_3.6.3	lifecycle_1.0.4
[139] bluster_1.12.0	lava_1.7.2.1	backports_1.5.0
[142] Brobdingnag_1.2-9	BiocParallel_1.36.0	timechange_0.2.0
[145] gtable_0.3.5	rjson_0.2.23	pROC_1.18.4
[148] jsonlite_1.8.9	colourpicker_1.3.0	bitops_1.0-8
[151] bit64_4.5.2	Rtsne_0.17	yulab.utils_0.1.7
[154] vegan_2.6-8	BiocNeighbors_1.20.2	RcppParallel_5.1.9
[157] bridgesampling_1.1-2	loo_2.6.0	shinyjs_2.1.0
[160] distributional_0.3.2	timeDate_4022.108	lazyeval_0.2.2
[163] pander_0.6.5	shiny_1.7.5.1	htmltools_0.5.8.1
[166] glue_1.7.0	RCurl_1.98-1.16	treeio_1.26.0
[169] gridExtra_2.3	boot_1.3-28.1	igraph_2.0.3
[172] R6_2.5.1	labeling_0.4.3	cluster_2.1.4
[175] pkgload_1.3.3	Rhdf5lib_1.22.1	aplot_0.2.2
[178] ipred_0.9-14	nloptr_2.1.1	DirichletMultinomial_1.44.0
[181] rstantools_2.3.1.1	DelayedArray_0.28.0	tidyselect_1.2.1
[184] vipor_0.4.7	htmlTable_2.4.3	microbiome_1.22.0
[187] tensorA_0.36.2	inline_0.3.19	ggforce_0.4.1
[190] car_3.1-2	future_1.33.0	ModelMetrics_1.2.2.2
[193] rsvd_1.0.5	munsell_0.5.1	dygraphs_1.1.1.6
[196] htmlwidgets_1.6.4	rlang_1.1.4	remotes_2.4.2.1
[199] ggnewscale_0.4.9	fansi_1.0.6	hardhat_1.3.0
[202] beeswarm_0.4.0	prodlim_2023.08.28	

For manuscripts utilizing custom algorithms or software that are central to the research but not yet described in published literature, software must be made available to editors and reviewers. We strongly encourage code deposition in a community repository (e.g. GitHub). See the Nature Portfolio guidelines for submitting code & software for further information.

Data

Policy information about availability of data

All manuscripts must include a data availability statement. This statement should provide the following information, where applicable:

- Accession codes, unique identifiers, or web links for publicly available datasets
- A description of any restrictions on data availability
- For clinical datasets or third party data, please ensure that the statement adheres to our policy

The metagenomic data are available from the European Genome-Phenome Archive (accession number EGAD00001007035). The phenotype data contain sensitive information from healthcare registers and are available through the THL biobank upon submission of a research plan and signing a data transfer agreement (https://thl.fi/en/web/thl-biobank/for-researchers/application-process).
The analysis source code is available for review as a tar archive. The code will be made public in a GitHub repository with a permanent DOI upon acceptance.

Research involving human participants, their data, or biological material

Policy information about studies with human participants or human data. See also policy information about sex, gender (identity/presentation), and sexual orientation and race, ethnicity and racism.

Reporting on sex and gender	Participant sex was identified using the social security number.
Reporting on race, ethnicity, or other socially relevant groupings	Household income data was collected based on a questionnaire and was used as the primary demographic descriptor variable alongside sex and age. We also used education level (educational years adjusted for birth year, with the levels low, medium, and high) in the models.
Population characteristics	No ethnicity data was collected from the participants. In 2002, the Finnish population was approximately 98% ethnic Finns and most foreigners were from Russia, Sweden and Estonia34. In FINRISK, 1.3% of the participants did not have Finnish or Swedish as their first language. Participant sex was identified using the social security number. Due to a lack of external cohorts with sufficient microbiome profiling and long-term health data, we used two internal subsamples to achieve a 70/30 train-test split (n = 5,000 and n = 2,098). We used cross-validation to examine the robustness of the results within the cohort.<br><br>Population density<br>The address-level coordinates of the participants were mapped with the sf R package 55 v. 1.0.9. to a 1 km2 population grid from 2005, obtained through the geofi R package 56 v. 1.0.7. of the participants' home addresses ranged from 1 to 19,175 inhabitants/km2 (mean 1,753 inhabitants/km2). The most densely populated regions are in Southern and South-Western Finland (in the cities of Helsinki and Turku, respectively). We classified the population density into five levels: (<10) 0-9 inhabitants/km2; (<100) 10-99; (<1,000) 100-999; (<10,000) 1000-9999; (<20,000) 10,000-20,000. The data points were randomly displaced within a 5 km x 5 km grid to obscure identifiable addresses in the figures. The figures do not show addresses with a population density of less than 10/km2.<br>Cumulative number of total antibiotic drug purchases during the past seven years before baseline varied from 0 to 85 (mean

3.3; Supplementary Fig. 1).

Baseline examination
The FINRISK 2002 survey included a self-administered questionnaire, physical measurements, and blood and stool sample collection. The questionnaire and an invitation to the health examination were mailed to all subjects. Trained nurses conducted physical examinations and blood sampling in local health centers or other survey sites. The participants were advised to fast for ≥4 hours and avoid heavy meals earlier during the day. The venous blood samples were centrifuged at the field survey sites, stored at −70°C, and transferred daily to the Finnish Institute for Health and Welfare laboratory. Data was collected for physiological measures, biomarkers, and dietary, demographic, and lifestyle factors.

Recruitment	The FINRISK population surveys were conducted every five years from 1972 to 2012 with the primary objective of tracking trends in cardiovascular disease risk factors in the Finnish adult population. The FINRISK 2002 study utilized a stratified random sampling approach of individuals between the ages of 25 and 74 from specific regions of Finland (Supplementary Fig. 1). These areas included North Karelia in the east, Northern Savonia in the east, Oulu in the northwest, the province of Lapland in the north, Turku and Loimaa regions in the southwest, and the cities of Helsinki and Vantaa capital region in the south. In addition, we used the West-East split of the regions based on the broad demographic and genetic characteristics of the Finnish population; the Western subset covers the regions of Turku/Loimaa and Helsinki/Vantaa, and the Eastern subset covers the rest of the regions (North Karelia, Northern Savonia, Oulu, Lapland). The sampling procedure was stratified by sex, region, and 10-year age group, resulting in 250 participants in each stratum. For Northern Karelia, Lapland, and the cities of Helsinki and Vantaa, the strata of 65-74-year-old men and women were also sampled, each with 250 participants. The initial population sample comprised 13,500 individuals (excluding 64 who had died or moved away between sample selection and the survey), with an overall participation rate of 65.5% (n=8,798). Of the participants, n=7,231 individuals successfully underwent stool shotgun sequencing. Of those,129 participants withdrew their consent from the THL Biobank at the time of the study. We excluded four individuals due to failed sequencing (< 100 reads). Subsequently, n=7,095 participants (mean age 49 years, 55 % women) remained for unsupervised analysis. The participants received no compensation.
Ethics oversight	The study protocol of FINRISK 2002 was approved by the Coordinating Ethical Committee of the Helsinki and Uusimaa Hospital District (Ref. 558/E3/20 1 All participants signed informed consent. The study was conducted according to the World Medical Association's Declaration of Helsinki on ethical principles.

Note that full information on the approval of the study protocol must also be provided in the manuscript.

Field-specific reporting

Please select the one below that is the best fit for your research. If you are not sure, read the appropriate sections before making your selection.

☒ Life sciences ☐ Behavioural & social sciences ☐ Ecological, evolutionary & environmental sciences

For a reference copy of the document with all sections, see nature.com/documents/nr-reporting-summary-flat.pdf

Life sciences study design

All studies must disclose on these points even when the disclosure is negative.

Sample size	The FINRISK population surveys were conducted every five years from 1972 to 2012 with the primary objective of tracking trends in cardiovascular disease risk factors in the Finnish adult population. The FINRISK 2002 study utilized a stratified random sampling approach of individuals between the ages of 25 and 74 from specific regions of Finland (Supplementary Fig. 1). These areas included North Karelia in the east, Northern Savonia in the east, Oulu in the northwest, the province of Lapland in the north, Turku and Loimaa regions in the southwest, and the cities of Helsinki and Vantaa capital region in the south. In addition, we used the West-East split of the regions based on the broad demographic and genetic characteristics of the Finnish population; the Western subset covers the regions of Turku/Loimaa and Helsinki/Vantaa, and the Eastern subset covers the rest of the regions (North Karelia, Northern Savonia, Oulu, Lapland). The sampling procedure was stratified by sex, region, and 10-year age group, resulting in 250 participants in each stratum. For Northern Karelia, Lapland, and the cities of Helsinki and Vantaa, the strata of 65-74-year-old men and women were also sampled, each with 250 participants. The initial population sample comprised 13,500 individuals (excluding 64 who had died or moved away between sample selection and the survey), with an overall participation rate of 65.5% (n=8,798). Of the participants, n=7,231 individuals successfully underwent stool shotgun sequencing. Of those,129 participants withdrew their consent from the THL Biobank at the time of the study. We excluded four individuals due to failed sequencing (< 100 reads). Subsequently, n=7,095 participants (mean age 49 years, 55 % women) remained for unsupervised analysis. We did not perform power analyses prior to the study, all participants with succesful sequencing data were included
Data exclusions	Of the participants, n=7,231 individuals successfully underwent stool shotgun sequencing. Of those,129 participants withdrew their consent from the THL Biobank at the time of the study. We excluded four individuals due to failed sequencing (< 100 reads). Subsequently, n=7,095 participants (mean age 49 years, 55 % women) remained for unsupervised analysis.
Replication	We split the participants in train and test sets 70/30 and replicated the models in test data.
Randomization	The split to test and train data was random. No further randomisation was done as this does not apply to population cohort studies.
Blinding	Blinding is not relevant to popualtion cohort studies.

Reporting for specific materials, systems and methods

We require information from authors about some types of materials, experimental systems and methods used in many studies. Here, indicate whether each material, system or method listed is relevant to your study. If you are not sure if a list item applies to your research, read the appropriate section before selecting a response.

Materials & experimental systems

n/a	Involved in the study
☒	☐ Antibodies
☒	☐ Eukaryotic cell lines
☒	☐ Palaeontology and archaeology
☒	☐ Animals and other organisms
☒	☐ Clinical data
☒	☐ Dual use research of concern
☒	☐ Plants

Methods

n/a	Involved in the study
☒	☐ ChIP-seq
☒	☐ Flow cytometry
☒	☐ MRI-based neuroimaging

Plants

Seed stocks	*Report on the source of all seed stocks or other plant material used. If applicable, state the seed stock centre and catalogue number. If plant specimens were collected from the field, describe the collection location, date and sampling procedures.*
Novel plant genotypes	*Describe the methods by which all novel plant genotypes were produced. This includes those generated by transgenic approaches, gene editing, chemical/radiation-based mutagenesis and hybridization. For transgenic lines, describe the transformation method, the number of independent lines analyzed and the generation upon which experiments were performed. For gene-edited lines, describe the editor used, the endogenous sequence targeted for editing, the targeting guide RNA sequence (if applicable) and how the editor was applied.*
Authentication	*Describe any authentication procedures for each seed stock used or novel genotype generated. Describe any experiments used to assess the effect of a mutation and, where applicable, how potential secondary effects (e.g. second site T-DNA insertions, mosiacism, off-target gene editing) were examined.*
